# Bone marrow stromal cell antigen-1 (CD157) regulated by sphingosine kinase 2 mediates kidney fibrosis

**DOI:** 10.3389/fmed.2022.993698

**Published:** 2022-10-04

**Authors:** Tsuyoshi Inoue, Yasuna Nakamura, Shinji Tanaka, Takahide Kohro, Lisa X. Li, Liping Huang, Junlan Yao, Suzuka Kawamura, Reiko Inoue, Hiroshi Nishi, Daichi Fukaya, Rie Uni, Sho Hasegawa, Reiko Inagi, Ryusuke Umene, Chia-Hsien Wu, Hong Ye, Amandeep Bajwa, Diane L. Rosin, Katsuhiko Ishihara, Masaomi Nangaku, Youichiro Wada, Mark D. Okusa

**Affiliations:** ^1^Division of Nephrology and Center for Immunity, Inflammation, and Regenerative Medicine, University of Virginia, Charlottesville, VA, United States; ^2^Department of Physiology of Visceral Function and Body Fluid, Graduate School of Biomedical Sciences, Nagasaki University, Nagasaki, Japan; ^3^Department of Clinical Informatics/Cardiology, Jichi Medical University, Tochigi, Japan; ^4^Division of Nephrology and Endocrinology, Graduate School of Medicine, The University of Tokyo, Tokyo, Japan; ^5^Division of Chronic Kidney Disease Pathophysiology, Graduate School of Medicine, The University of Tokyo, Tokyo, Japan; ^6^Department of Pharmacology, University of Virginia, Charlottesville, VA, United States; ^7^Department of Immunology and Molecular Genetics, Kawasaki Medical School, Okayama, Japan; ^8^Isotope Science Center, The University of Tokyo, Tokyo, Japan

**Keywords:** BST-1/CD157, kidney, fibrosis, ADP-ribosyl cyclase, GPI-linked proteins

## Abstract

Chronic kidney disease is a progressive disease that may lead to end-stage renal disease. Interstitial fibrosis develops as the disease progresses. Therapies that focus on fibrosis to delay or reverse progressive renal failure are limited. We and others showed that sphingosine kinase 2-deficient mice (*Sphk2*^–/–^) develop less fibrosis in mouse models of kidney fibrosis. Sphingosine kinase2 (SphK2), one of two sphingosine kinases that produce sphingosine 1-phosphate (S1P), is primarily located in the nucleus. S1P produced by SphK2 inhibits histone deacetylase (HDAC) and changes histone acetylation status, which can lead to altered target gene expression. We hypothesized that Sphk2 epigenetically regulates downstream genes to induce fibrosis, and we performed a comprehensive analysis using the combination of RNA-seq and ChIP-seq. Bst1/CD157 was identified as a gene that is regulated by SphK2 through a change in histone acetylation level, and *Bst1*^–/–^ mice were found to develop less renal fibrosis after unilateral ischemia-reperfusion injury, a mouse model of kidney fibrosis. Although Bst1 is a cell-surface molecule that has a wide variety of functions through its varied enzymatic activities and downstream intracellular signaling pathways, no studies on the role of Bst1 in kidney diseases have been reported previously. In the current study, we demonstrated that Bst1 is a gene that is regulated by SphK2 through epigenetic change and is critical in kidney fibrosis.

## Introduction

Acute kidney injury (AKI), a potentially fatal disorder (1–3) can lead to chronic kidney disease (CKD) with progression to end-stage renal disease requiring dialysis or kidney transplantation (4, 5). CKD has other causes, such as primary glomerular disease, diabetes mellitus, hypertension, and aging, and its prevalence is increasing. CKD is accompanied by fibrosis regardless of the underlying cause, and fibrosis is irreversible (3, 6).

Sphingosine is derived from plasma membrane lipids and is phosphorylated by sphingosine kinase 1 (SphK1) and SphK2 to produce sphingosine 1-phosphate (S1P). Extracellular S1P, acting through its five G protein-coupled receptor subtypes (S1P1 to S1P5), exerts various functions such as vascular development and immune cell trafficking (7). We have focused on the function of S1P and its receptors in the kidney and have revealed a protective role for proximal tubule S1P1 (8) and endothelial cell S1P1 (9) and a detrimental role of dendritic cell S1P3 (6, 10, 11) in AKI.

SphK2 is located predominantly in the nucleus (12), in contrast to SphK1, which is largely cytoplasmic and produces S1P that acts primarily at S1P receptors (13, 14). S1P generated by SphK2 binds to and inhibits histone deacetylase (HDAC) activity (15). HDAC removes acetyl groups from histones, leading to a reduced histone acetylated status. Inhibition of HDAC, which enhances histone acetylation around genes, induces chromatin relaxation thereby enabling enhanced gene expression (16). Thus, SphK2-produced S1P induces gene expression of SphK2-regulated genes through epigenetic changes. In the absence of SphK2 in *Sphk2*^–/–^ mice, HDAC activity is enhanced. This causes less histone acetylation around SphK2-regulated genes, which reduces their expression. We and others have shown that *Sphk2*^–/–^ mice develop less fibrosis than WT or *Sphk1*^–/–^ mice in folic acid-induced or unilateral ischemia-reperfusion injury (IRI)-induced kidney fibrosis models (17) and in the unilateral ureteral obstruction-induced model of kidney fibrosis (18), implying that expression of fibrosis-related genes is also suppressed. Considering that *Sphk2*^–/–^ mice are protected from kidney fibrosis following injury and that SphK2 regulates histone acetylation status, we hypothesized that there are genes that induce fibrosis through SphK2-induced histone acetylation. Our analysis using RNA-seq and ChIP-seq of gene regulation and histone acetylation at histone 3 lysine 9 (H3K9ac) and histone 3 lysine 27 (H3K27ac) identified genes regulated by SphK2, and we found that one of these genes, *Bst1* also known as CD157, is important in the progression of kidney fibrosis.

## Results

**FIGURE 1:**
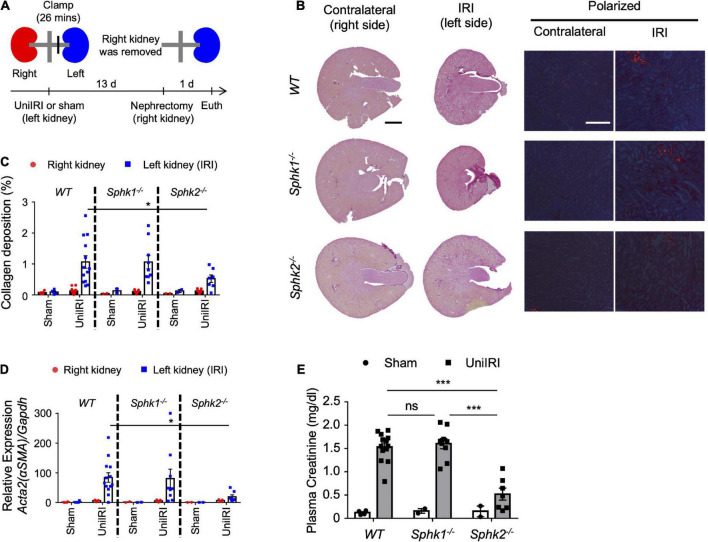
Sphk2-deficient mice develop less renal fibrosis. **(A)** Experimental plan for b-e. (b-e) Nephrectomy (right; uninjured kidney) 13 days after renal unilateral (left) ischemia-reperfusion injury (UniIRI; 26 min ischemia); kidney injury evaluated 1 day later. **(B)** Representative photographs of picrosirius red staining of kidney sections. Interstitial collagen deposition is expressed as the percentage of the kidney tissue section surface area occupied by red/yellow pixels detected under polarized light microscopy. *Sphk2 ^–/–^* develop less renal fibrosis as shown by tissue morphology **(C)**, scored from polarized picrosirius red photographs in panel **(B)**; % of kidney cross-sectional area) and RT-PCR of fibrosis markers [**(D)** RNA from whole kidney], and their kidney function is preserved **(E)** as indicated by reduced plasma creatinine. *n* = 7–13 in panels **(B–E)**. Data were analyzed using one-way ANOVA in panel **(E)** and two-way ANOVA in panels **(C,D)** followed by *post hoc* multiple-comparison test (Tukey’s). *P < 0.05 and ***P < 0.001. In this and subsequent figures, only statistically significant differences of interest are marked. Scale bar = 1 mm in whole kidney, 200 μm in polarized view in panel **(B)**.

Using a unilateral IRI (uniIRI) model of fibrosis, as shown in Figure 1A, we showed that *Sphk2*^–/–^ mice develop less renal fibrosis. At day 14 following uniIRI, kidneys of *Sphk2*^–/–^ mice had less collagen deposition as shown by picrosirius red staining (Figure 1C and representative photographs in Figure 1B) and reduced expression of *Acta2* (αSMA), a fibrosis-related gene (Figure 1D), compared to WT or *Sphk1*^–/–^ mice, which confirms previous findings in 2 models of kidney fibrosis (17). Nephrectomy of the right (uninjured) kidney was performed on day 13, and plasma creatinine was evaluated on day 14 as a measure of function of the remaining injured kidney (left). The increase in plasma creatinine observed in WT and *Sphk1*^–/–^ mice on day 14 was suppressed in *Sphk2*^–/–^ mice, indicating that kidney function was preserved in *Sphk2*^–/–^ mice after uniIRI (Figure 1E). Maintenance of renal function 14 days after unilateral IRI was not observed in heterozygotes (*Sphk2*^+/–^) (Supplementary Figures 1, 2).

**FIGURE 2:**
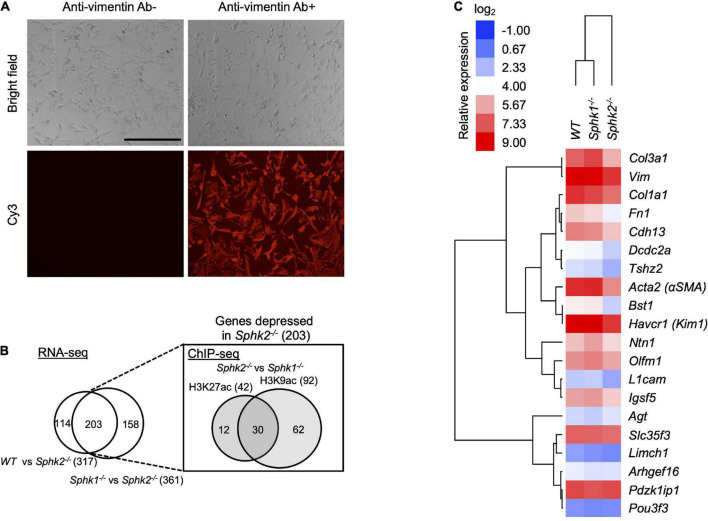
Genome-wide analysis and *in vivo* screening identified *Bst1* as a gene related with kidney fibrosis. **(A)** Characteristics of primary renal fibroblasts. Fibroblasts isolated from kidneys of untreated, unoperated mice, were cultured and passages 4–10 were used for all experiments. Immunocytochemistry was performed to confirm that the isolated cells were fibroblasts. An elongated, spindle-shaped morphology (phase contrast, top panels) and strong vimentin staining (red immunofluorescence, bottom panels) were observed. Anti-Vimentin Ab + : anti-vimentin, 1:250, rabbit monoclonal, ab92547, Abcam, followed by donkey anti-rabbit-Cy3, 1:500, Jackson ImmunoResearch; Anti-Vimentin Ab-: primary antibody omitted. Scale bar = 400 μm. **(B)** Venn diagram based on RNA-seq and ChIP-seq of primary renal fibroblasts isolated from kidneys of untreated, unoperated *WT*, *Sphk1*^–/–^ and *Sphk2*^–/–^ mice. From 203 genes identified by RNA-seq that have suppressed expression in *Sphk2*^–/–^ cells (the overlap of comparison of *Sphk2*^–/–^ cells to WT and *Sphk1*^–/–^cells, left), 30 genes that have lower histone acetylation at two sites (lysine 27 and lysine 9) on histone 3 (H3K27 and H3K9, respectively) in *Sphk2*^–/–^ cells were chosen by ChIP-seq (the overlap of comparison of *Sphk1*^–/–^ and *Sphk2*^–/–^ cells, right). **(C)** *In vivo* screening. Heat map based on expression analysis of common fibrosis-related genes and the top 50% of selected genes in panel **(A)** (RT-PCR of RNA isolated from kidneys after unilateral IRI). Clustering was performed based on relative gene expression. Relative expression was calculated as the ratio of gene expression in the left kidney (IRI side) compared to the right kidney (non-IRI side) of wild-type mice. *n* = 5–7 in panel **(C)**. Gene abbreviations defined in Supplementary Table 3 and Supplementary Figure 3.

To identify genes regulated by SphK2, we performed analysis by RNA and chromatin immunoprecipitation sequencing (RNA-seq and ChIP-seq, respectively) using primary renal fibroblasts isolated from kidneys of unoperated *Sphk2*^+/+^(WT), *Sphk1*^–/–^ and *Sphk2*^–/–^ mice. Characteristics of fibroblasts are shown in Figure 2A and an elongated, spindle-shaped morphology and strong vimentin staining were observed. RNA-seq analysis identified 203 genes in *Sphk2*^–/–^ mice that were down-regulated (with expression level < 1/4) compared to *Sphk2*^+/+^ and *Sphk1*^–/–^ mice (the overlap of 317 genes identified in a WT vs. *Sphk2*^–/–^ comparison with 361 genes identified in a *Sphk1*^–/–^ and *Sphk2*^–/–^ comparison; Figure 2B, left). We paid particular attention to differences in these 203 suppressed genes and compared their histone acetylation status in fibroblasts derived from *Sphk1*^–/–^ and *Sphk2*^–/–^ using ChIP-seq. We analyzed differences in histone acetylation at 2 acetylation sites—acetylated histone 3 lysine 9 (H3K9ac) and acetylated histone 3 lysine 27 (H3K27ac), because these are two of the most commonly studied sites (19). ChIP-seq (summary data in Supplementary Table 1) revealed suppressed acetylation in 42 genes at H3K27 and in 92 genes at H3K9 only in *Sphk2*^–/–^ and not in *Sphk1*^–/–^; the overlap in comparison of *Sphk1*^–/–^ and *Sphk2*^–/–^ fibroblasts shows 30 genes that have lower histone acetylation at these two sites (Figure 2B, right). Thus, the combination of RNA-seq and ChIP-seq analysis yielded 30 candidate genes that might be regulated by SphK2 through a change in histone acetylation status (Supplementary Table 2).

To investigate fibrotic function of the selected candidate genes by *in vivo* screening, unilateral IRI (uniIRI) was used as a kidney fibrosis model (the same protocol was applied as Figure 1A). To examine the expression pattern of the selected genes, real time PCR was performed on day 14 on the kidney subjected to uniIRI. Expression of fibrosis-related genes, including *Acta2* (αSMA), *Col3a1*, *Col3a3*, *Vim* and *Fn1*, was induced by uniIRI in WT and *Sphk1*^–/–^ mice but was suppressed in *Sphk2*^–/–^ mice (Figure 2C and Supplementary Figure 3). Some of the genes, such as *Bst1* and *Havcr1* (Kim1), which were among the genes selected by the genome-wide analysis, showed a similar expression pattern in the kidney as these known fibrosis-induced genes (Figure 2C and Supplementary Figure 3). This suggests that *Bst1* and/or *Havcr1* (*Kim1*) might be genes related with fibrosis. However, because Havcr1 (Kim1) is well known as a kidney injury marker and its role has been extensively studied (20, 21), we focused on Bst1 for further analysis.

**FIGURE 3:**
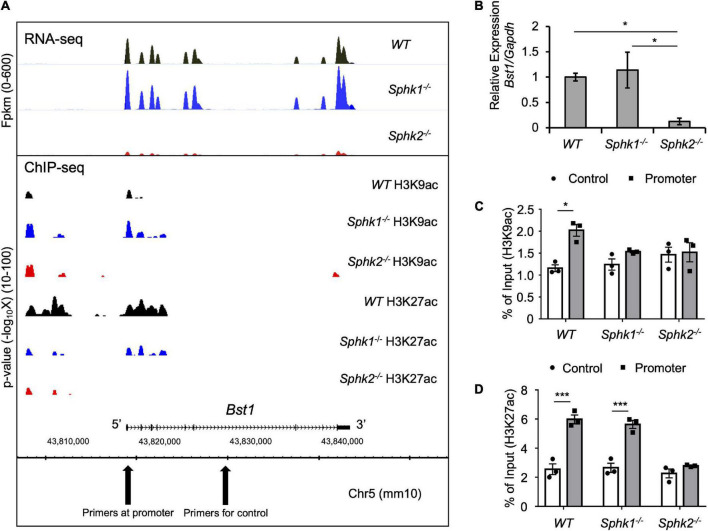
Genome-wide analysis data around *Bst1* gene. **(A)** RNA-seq and ChIP-seq were performed using RNA or DNA of fibroblasts isolated from kidneys of unoperated *WT*, *Sphk1*^–/–^ and *Sphk2*^–/–^ mice and showed suppressed gene transcription (top panel) and histone acetylation around *Bst1* gene (bottom panel) in *Sphk2*^–/–^-derived cells. X-axis shows the locus of the genome. Top panel, Bst1 gene expression as Fpkm (fragments per kilobase of exon per million mapped fragments) across the genome including the *Bst1* gene. Bottom panel: Localization and magnitude of histone marks on the genome around *Bst1* gene. Y axis shows the statistical significance (*p* value) of the enrichment level of ChIP signals in the regions comparing to background. **(B)** Real time PCR of *Bst1* confirmed RNA-seq data. ChIP-qPCR (expressed as% of input DNA) of H3K9ac **(C)** and H3K27ac **(D)** confirmed ChIP-seq data. *n* = 4 in panel **(B)**. *n* = 3 in panels **(C,D)**. Data were analyzed using one-way ANOVA in panel **(B)** and two-way ANOVA in panels **(C,D)** followed by *post hoc* multiple-comparison test (Tukey’s). * *P* < 0.05 and *** *P* < 0.001.

**FIGURE 4:**
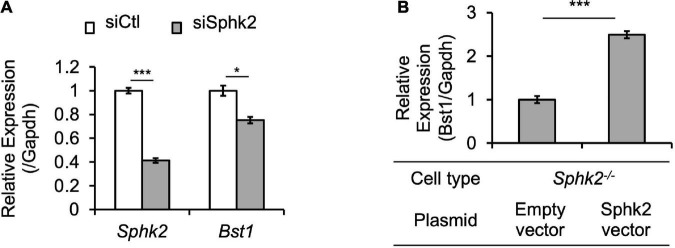
SphK2 regulates *Bst1* expression. **(A)** si*Sphk2* suppresses *Bst1* expression in *Sphk2*^+/+^ fibroblasts. **(B)** SphK2 overexpression rescues *Bst1* expression in *Sphk2*^–/–^ fibroblasts. *n* = 3 each in panels **(A,B)**. Data in panels **(A,B)** were analyzed with Student’s *t*-test (2 tailed). * *P* < 0.05 and *** *P* < 0.001. siCtl, control siRNA.

Bst1 is a cell-surface molecule that has a wide variety of functions, through its varied enzymatic activities and downstream intracellular signaling pathways, including modulation of immune response (22). Bst1 has been associated with diseases, such as ovarian cancer (23), Parkinson’s disease (24), and rheumatoid arthritis (25). RNA-seq and ChIP-seq data showed that gene transcription and histone acetylation around the *Bst1* gene are suppressed in *Sphk2*^–/–^ mice (Figure 3A). ChIP-qPCR in addition to real time PCR (Figures 3C,D; with further confirmation of reduced expression of *Bst1* by qPCR in Figure 3B) confirmed that histone acetylation level at the promoter region of *Bst1* and gene expression of Bst1 were suppressed in *Sphk2*^–/–^-derived fibroblasts. SphK2 knock down in *Sphk2*^+/+^ fibroblasts suppressed *Bst1* expression (Figure 4A) and SphK2 overexpression (Figure 4B) in fibroblasts from *Sphk2*^–/–^ mice rescued Bst1 expression, showing that *Bst1* gene expression is regulated by SphK2.

**FIGURE 5:**
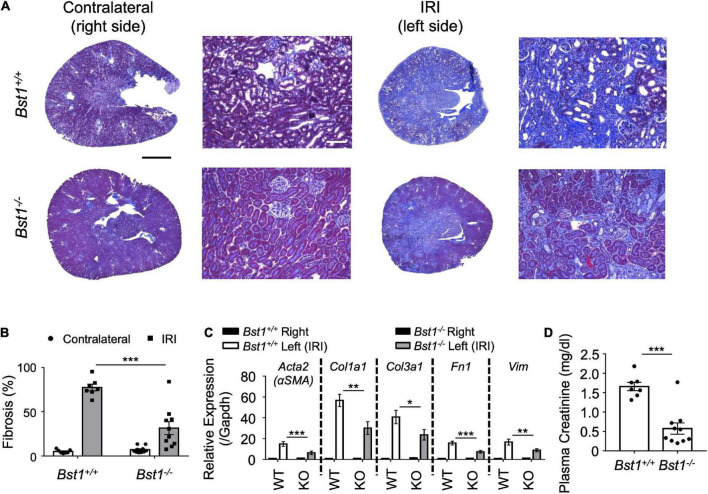
*Bst1*-deficient mice develop less renal fibrosis. Nephrectomy (right, unoperated kidney) was performed 13 days after renal unilateral (left, ischemic kidney) ischemia-reperfusion injury (IRI; 26 min ischemia; as in Figure 1A). One day later the extent of kidney injury was evaluated. **(A,B)** Representative images and quantification of kidney sections by Masson’s trichrome staining. The fibrotic area is expressed as the percentage of the kidney tissue section surface area occupied by blue trichrome stain. *Bst1*-deficient mice develop less renal fibrosis as shown by tissue morphology [**(A)** representative Masson’s trichrome staining of kidney sections, **(B)** scored from Masson’s trichrome stained sections] and RT-PCR of fibrosis markers [**(C)** RNA was isolated from whole kidney]. WT, *Bst1*^+/+^ mice; KO, *Bst1*^–/–^ mice. **(D)** Kidney function of the injured (fibrotic) kidney (left) was evaluated by plasma creatinine 1 day after contralateral (right) nephrectomy. Kidney function was preserved in *Bst1*^–/–^ mice after unilateral IRI. *n* = 7–10 in panels **(B–D)**. Data in panels **(B,C)** were analyzed with two-way ANOVA. Means were compared by *post hoc* multiple-comparison test (Tukey’s). Data in panel **(D)** were analyzed with Student’s *t*-test (2 tailed). * *P* < 0.05, ** *P* < 0.01 and *** *P* < 0.001. Scale bar = 1 mm in whole kidney and 200 μm in inset.

To evaluate the role of Bst1 in the context of fibrosis, we subjected *Bst1*^–/–^ mice to unilateral IRI, the same method applied to *Sphk2*^–/–^ mice, and found that *Bst1*^–/–^ mice developed less renal fibrosis than WT controls (Figures 5A,B and Supplementary Figures 4, 5). Expression of fibrosis-related genes was suppressed and the function of injured kidney was preserved in *Bst1*^–/–^ mice (Figures 5C,D and Supplementary Figures 6, 7). These data indicate that *Bst1* is a gene that is associated with fibrosis, as we expected.

## Discussion

The essential new findings are as follows. The combination of genome wide analysis and *in vivo* screening identified *Bst1/CD157* as a gene that is regulated by SphK2 through histone acetylation. Modifications of Sphk2 expression including *SphK2* knock down and *SphK2* overexpression experiments revealed that *Bst1* gene expression is regulated by SphK2. We revealed that *Bst1* is critical in the development of renal fibrosis.

### Bst1 as a new downstream target of SphK2

*Sphk2*^–/–^ mice develop less renal fibrosis than *Sphk1*^–/–^ mice (Figures 1B–E), and S1P and SphK2 are part of a corepressor complex that influences histone acetylation and gene expression (15, 26); therefore we focused on identifying targets downstream of SphK2. A variety of cell types (e.g., kidney tubule epithelial cells, immune cells, endothelial cells) participate in kidney injury and fibrosis, but we focused our attention first on factors in fibroblasts that could contribute to fibrosis owing to their central role. RNA-seq identified the genes that have lower expression in *Sphk2*^–/–^-derived fibroblasts, and ChIP-seq on histone acetylation found the genes that have lower histone acetylation status around their promoter regions in *Sphk2*^–/–^-derived fibroblasts compared to *Sphk2*^+/+^ (WT) or *Sphk1*^–/–^-derived fibroblasts. We identified Bst1 as a new downstream target of SphK2 (Figures 2–4). There are other candidate genes that may be downstream of SphK2 as listed in Supplementary Table 2. Some of the genes also might have functions associated with fibrosis, especially the genes showing similar expression pattern as Bst1 and other fibrosis-related markers (Figure 2C) and will be the subject of future studies. HDACs can also deacetylate numerous non-histone proteins including transcription factors such as signal transducer and activator of transcription 1 (Stat1), Stat3, NF−κB and forkhead box P3 (Foxp3) (27), implying that downstream genes might be regulated by SphK2 through the change of acetylation on non-histone proteins. Further analysis will be needed to reveal the importance of these other genes in fibrosis and the differences between the roles of SphK1 and SphK2. In addition, although ChIP-seq data showed H3K9ac around Bst1 in wild-type and *Sphk1*^–/–^ mice, ChIP-qPCR showed that acetylation of H3K9ac around Bst1 gene was suppressed in *Sphk1*^–/–^ mice (Figures 3A,C). The location of histone tail acetylation, its relationship to gene expression levels, and its association with renal injury remain largely unknown and will require further investigation.

### Bst1 is a multi-functional molecule

Bst1/CD157 was initially identified as a surface molecule of stromal cells involved in pre-B lymphocyte growth (25). This molecule also belongs to the ADP-ribosyl cyclase family that plays an important role in Ca^2+^ homeostasis (22, 28). Bst1 is an immunoregulatory molecule and could impact the innate response of kidney injury and serve as a potential therapeutic target (29). Bst1 is expressed widely in immune cells including bone marrow precursor cells (30) and leukocytes including neutrophils (31), basophils (32), monocytes (32), macrophages (33) and plasmacytoid dendritic cells (34). Furthermore, Bst1 is expressed in endothelial cells, cells that are important in mediating leukocyte migration and Bst1 appears to play a key role in leukocyte recruitment to the site of inflammation (35). Anti-CD157 and anti-CD31 mAb were used to block neutrophil interaction with the endothelium which led to the loss of diapedesis. These results and other findings indicate that Bst1 is a key player in the control of leukocyte adhesion, migration and diapedesis *via* phosphorylation of Akt and MAPKs (31, 35, 36). Other functions of Bst1 such as its roles in humoral immune response (37), regulation of intestinal homeostasis (38), regulation of behaviors related with depression and anxiety (39, 40) have been revealed. The links between CD157 and pathological conditions were reported in rheumatoid arthritis (41), acute myeloid leukemia (42–44), ovarian cancer (44), malignant mesothelioma (45) and Parkinson’s disease (46). In addition, scrapie responsive gene 1 (Scrg1) was identified as a ligand for Bst1 regulating stemness of mesenchymal stem cells (47).

### A new role of Bst1 in kidney injury

While various functions of Bst1 were revealed as described above, the function of Bst1 in the context of organ fibrosis has never been reported. Although the expression of Bst1 in the kidney was documented in a few studies, the role of Bst1 had never been evaluated (25, 48). One paper showed that another ADP ribosyl cyclase, CD38, which is not Bst1, plays an important role in acute vascular responses to angiotensin II, endothelin-1, and norepinephrine (45, 49). The current study clearly showed a new role for Bst1 in uniIRI-induced kidney fibrosis using Bst1-deficient mice (Figure 5). It will be necessary to verify whether Bst1-deficient mice develop less fibrosis in other models of kidney fibrosis (e.g., folic acid-induced or unilateral ureteral obstruction-induced model of kidney fibrosis). The public single cell datasets from the Humphreys’ lab^1^ suggests that Bst1 is abundantly expressed in distal tubule and its expression is enhanced at 12 h after renal ischemia-reperfusion injury (50). Furthermore, there is a report that blocking Sphk2, which is highly expressed in the proximal tubules, improved cisplatin-induced nephropathy (51), suggesting that Bst1 may be related to other factors besides fibrosis.

There are some limitations to our study: the fibroblasts used to identify Bst1 were cultured primary cells and could have lost their *in vivo* identities. Also, since we used fibroblasts from kidneys of unoperated mice, histone modifications and gene expression status in the fibrotic state might be different than in the unoperated state. In addition, we could not confirm whether Bst1 is expressed in fibroblasts, tubules, immune cells, or endothelial cells in the kidney, and how it is altered by injury in this study. Bst1 research in the kidney has just begun and further research is needed on the localization of Bst1 in the kidney and the molecular mechanism of how Bst1 causes renal fibrosis. Recently Bst1 was identified as a marker of tissue-resident vascular endothelial stem cells in various arteries and veins of mouse organs, and single-cell RNA-seq technology was also applied in Bst1 research (52, 53). Utilizing these technologies, it is expected that the elucidation of the function of Bst1 in the kidney will be accelerated.

In summary, we have identified Bst1 as a downstream target of Sphk2 that has a previously unrecognized role in kidney injury and revealed that Bst1 deficiency leads to less renal fibrosis. These findings uncovered a new pathway in kidney fibrosis that highlights Bst1 as a potential therapeutic target.

## Materials and methods

### Mice

Male mice (8–12 weeks of age, 20–25 g) were used for all experiments. *Sphk1*^–/–^ and *Sphk2*^–/–^ mice (congenic on C57BL/6) were provided by Dr. Richard L. Proia (National Institutes of Health/National Institute of Diabetes and Digestive and Kidney Diseases, Bethesda, MD, United States) and were crossed with C57BL/6 mice to generate WT littermates (see Supplementary Figures 1, 2). *Bst1*^–/–^ mice (congenic on C57BL/6) were provided by Dr. Katsuhiko Ishihara, a co-author (54), and were crossed with C57BL/6 mice to generate WT littermates (see Supplementary Figure 7). Wildtype littermates or progeny were used as controls for all the other experiments.

### Fibroblast isolation

Unoperated, untreated mice were euthanized, and dipped in 70% isopropanol, and the kidneys were removed. Kidneys were rinsed with sterile PBS and were minced. The kidney tissue suspension was gently pressed and rinsed through a 40-μm filter (Thermo Fisher Scientific) with sterile PBS twice using the rubber end of a 5-ml syringe plunger and then centrifuged at 500 *g* for 5 min at 4°C. After cell pellets were resuspended in 10 ml medium (DMEM, 10% FBS, 1% penicillin-streptomycin-amphotericin B), cells were plated in a 10 cm dish and incubated overnight allowing some cells to adhere. The next day the medium and non-adherent cells in the plate was collected, combined with 20 ml fresh media, and divided into three 10 cm dishes to allow for additional cells to adhere to the plate; fresh media was added to the original plate. When adherent cells became confluent, they were combined and passed to generate passage 1. Cells from passages 4–10 were used for experiments. Immunohistochemistry using vimentin as a marker for fibroblasts (55) was performed and all cells cultured were confirmed to be vimentin positive. The characteristics of the cells are shown in Figure 2A.

### Unilateral ischemia-reperfusion injury

Mice were anesthetized by the intraperitoneal administration of ketamine (120 mg/kg) and xylazine (12 mg/kg) and underwent renal ischemia-reperfusion injury (IRI), as previously described (56). Unilateral (left) kidney IRI was performed through flank incisions by clamping the kidney pedicle for 26 min. The clamp was then removed and the wound sutured after restoration of blood flow was visually observed. Sham-operated mice underwent the same procedure except that the kidney pedicle was not clamped. Nephrectomy (right; uninjured contralateral kidney) was performed 13 days after kidney IRI (contralateral kidney was saved for analysis) to reveal changes in kidney function (plasma creatinine) of the operated, ischemic (left) kidney. Mice were euthanized 24 h later, and kidney function was evaluated by measuring plasma creatinine.

### Plasma creatinine and histological analysis

Plasma was prepared by centrifuging heparinized blood at 7,000 × *g* for 5 min. Plasma creatinine (mg/dl) was measured by using an enzymatic method with minor modifications from the manufacturer’s protocol (using double the sample volume; Diazyme Laboratories). We previously validated the enzymatic kit by comparing with analysis of creatinine by liquid chromatography–mass spectrometry (LC-MS) (57).

Kidney fibrosis was evaluated as we previously reported (58) using Masson’s trichrome stain, which detects collagen in all fibrotic processes (but does not discriminate collagen subtypes), and picrosirius red stain, with polarized light microscopy to view birefringence of large mature type 1 collagen bundles that account for a portion of the total collagen deposition; smaller collagen bundles are weakly birefringent. Kidneys were dissected and the capsule was removed. A center transverse section was cut and placed in 4% PLP (4% paraformaldehyde/1.4% DL-lysine/0.2% sodium periodate in 0.1 M sodium phosphate buffer, pH 7.4) for 24 h and then stored in 70% EtOH until paraffin embedding (UVA Research Histology Core). Paraffin-embedded kidneys were cut into 5 μm sections that were then stained with Masson’s trichrome or picrosirius red. The sections were observed under light microscopy (Zeiss AxioImager Z1/Apotome microscope, Carl Zeiss Microscopy). Photographs were taken with an AxioCam MRc camera (Zeiss) and brightness/contrast and white balance adjustments were made using StereoInvestigator software (Version 2017; MBF Bioscience). The extent of kidney tubulointerstitial fibrosis (Masson’s trichrome staining) in the whole kidney section, but excluding papilla, was assessed in an unbiased, systematic manner using design-based stereology to achieve statistically accurate random sampling of kidney sections and yielding the percentage of total area of the section occupied by tubulointerstitial fibrosis (Masson’s trichrome staining), as described (17, 57). The investigator was blinded to the experimental identity of the sections. Sections were imaged by using a Zeiss AxioImager Z1/Apotome Microscope fitted with motorized focus drives and motorized XYZ microscope stage and integrated to a workstation running StereoInvestigator software. The area fraction fractionator probe was used for stereological analysis of the fractional area of the section occupied by tubular necrosis. The following parameters were defined: counting frame, 400 × 400 μm^2^; sample grid, 1,200 × 1,200 μm^2^ and grid spacing, 85 μm. These values were determined empirically such that adequate numbers of sample sites were visited and adequate numbers of markers (indicating areas of trichrome staining) were acquired, in keeping with accepted counting rules for stereology. A total of 120 ± 6.8 (mean ± SEM) grid sites was evaluated per section.

### Picrosirius red staining

Kidneys were fixed in 4% PLP for 24 h, changed to 70% ethanol, and subsequently embedded in paraffin and sectioned at 5 μm thickness. Sections were dewaxed in xylenes, rehydrated, and stained in picrosirius red solution (Electron Microscopy Services) for 1 h. After washing in acidified water, sections were dehydrated in three changes of 100% ethanol and cleared with xylene, and coverslips were applied. Stitched brightfield photos of whole kidney were captured using a Zeiss AxioImager microscope and StereoInvestigator software. Photomicrographs of picrosirius red birefringence were captured using a polarizing filter (Carl Zeiss GmbH). Quantification of the fibrotic area was done by measuring total red/yellow birefringent pixels per total kidney section surface area with ImageJ software^2^.

### RNA-sequencing

RNA was isolated from fibroblasts using the RNeasy Micro Kit (Qiagen). Poly(A)-containing mRNA molecules were isolated from total RNA, then converted to cDNA with poly A primers using a TruSeq RNA Sample Preparation kit v2 (Illumina). Sequenced paired-end reads were mapped onto the mouse genome build mm10 using TopHat version 2.0.13 (59) and the FPKM (fragments per kilobase of transcript per million mapped fragments) were calculated as gene expression level using Cufflinks version 2.2.1 (60) along with the default parameter settings (61). High-throughput sequencing for mRNA-seq was carried out using a Hiseq2500 (Illumina) system. For analysis and visualization of the data generated by Cufflinks, we used the R package cummeRbund^3^.

### Chromatin immunoprecipitation

ChIP assay was performed as previously described (62, 63). Two million fibroblasts were plated on a 10-cm culture plate and maintained in culture medium (DMEM, 10% FBS, 1% penicillin-streptomycin-amphotericin B). The cells were crosslinked for 10 min using 1% paraformaldehyde/0.1 M phosphate, pH 7.4 at the appropriate time thereafter. After neutralization using 0.2 M glycine, cells were collected, resuspended in SDS lysis buffer (10 mM Tris–HCl, 150 mM NaCl, 1% SDS, 1 mM EDTA; pH 8.0) and fragmented by sonication (Ultrasonic processor 130 W, amplitude 50%, turn on 1 s, turn off 1 s for 5 min; Cole Parmer). Samples were stored at −80°C before use. To perform ChIP, antibodies against H3K9ac (MA305B) and H3K27ac (MA309B; Takara Bio United States) were used in combination with magnetic beads (Thermo Fisher Scientific). Prepared DNA was quantified using Synergy HTX (BioTek Instruments) and more than 5 ng of DNA was processed for sequencing, as described below. Real time PCR (ChIP-qPCR) was performed using the obtained DNA and non-immunoprecipitated DNA (input DNA). Input DNA was used as a negative control to define non-specific binding, and data (Figures 3C,D) are expressed as% of input DNA. The ChIP primer sequences for negative control and *Bst1* promoter (and respective results shown as Control and Promoter in Figures 3C,D) are listed in Supplementary Table 4.

### ChIP-seq

ChIP-seq, or chromatin immunoprecipitation combined with DNA sequencing, was used to analyze protein interactions with DNA, in this case interactions of histones, identified by specific histone acetylation sites, associated with the Bst1 gene. All of the protocols for Illumina/Solexa sequence preparation, sequencing and quality control were provided by Illumina. Sequences were aligned using mouse genome build UCSC mm10 as the reference genome. Non-immunoprecipitated DNA (input DNA) was used as a negative control to define non-specific binding. All uniquely mapped sequences were analyzed by Quantitative Enrichment of Sequence Tags (QuEST) 2.4 software using the default parameters (KDE bandwidth = 100 bp, region size = 1,000 bp, ChIP seeding fold enrichment = 30, ChIP extension fold enrichment = 3, and ChIP-tobackground fold enrichment = 3) (64). WIG files were generated with QuEST, which were subsequently used for visualization purposes and for obtaining the average signal profiles. These signals were visualized using Integrated Genome Browser software^4^ with normalized profile wig files calculated by QuEST.

### Real-time PCR

RNA was isolated using RNeasy Mini plus kit (Qiagen), and RNA concentration was determined based on spectrophotometric determination of 260/280 ratio. cDNA was generated from the resultant RNA using the iScript cDNA synthesis kit (Bio-Rad) as described by the manufacturer. Resultant cDNA was then used to determine relative mRNA expression of various genes using the iTAC Universal SYBR Green Supermix (Bio-Rad). Primer sequences are shown in Supplementary Table 3. Relative gene expression was calculated from RT-PCR data as the ratio of expression in the left kidney (IRI side) compared to the right kidney (non-IRI side) of wild-type mice Based on these relative expression values, clustering was performed using Cluster 3.0 (65), and a heat map was created with Java TreeView 1.1 (66) (Figure 2C).

### Gene knockdown by siRNA

Fibroblasts were plated in a 24 well cell culture plate with 500 μl medium (DMEM, 10% FBS, 1% penicillin-streptomycin-amphotericin B) at a density of 2.0 × 10^5^ cells/ml and cultured overnight. The cells were transfected with ON-TARGET plus siRNA for SphK2 (Dharmacon, J-041258-0) or ON-TARGET plus Control siRNA #1 (Dharmacon, D-001818-01-20) at a concentration of 25 nM using TransIT-X2 reagent according to the manufacturer’s protocol. 24 h after transfection, the cells were used for further analysis. The knockdown efficiencies of SphK2 were validated by qRT-PCR (CFX96, BioRad) using the same primers described in Supplementary Table 3 (Figure 4A).

### Gene overexpression

Fibroblasts were plated in 24 well cell culture plate with 500 μl medium (DMEM, 10% FBS, 1% penicillin-streptomycin-amphotericin B) at a density of 2.0 × 10^5^ cells/ml and cultured overnight. The cells were transfected with SphK2 plasmid (SphK2-pcDNA) or empty-GFP plasmid (Thermo Fisher Scientific, pcDNA3.1) at a concentration of 1 μg/ml using TransIT-X2 reagent according to the manufacturer’s protocol. 24 h after transfection, the cells were used for further analysis. The overexpression was confirmed by fluorescence microscopy of native GFP fluorescence.

### Immunocytochemistry

One day after fibroblasts (1.5 × 10^5^ cells/well) were plated in a 24-well plate, the cells were washed with PBS × 3 times, then the cells were fixed with 100% methanol/0.1% Triton X-100 for 10 min at room temperature. After the cells were washed again with PBS × 3 times, the cells were blocked with DAKO Protein Block Serum-Free for 5 min at room temperature. After the blocking buffer was aspirated, cells were incubated overnight with primary antibody (anti-vimentin, 1:250, rabbit monoclonal, ab92547 Abcam) in 1% BSA/PBS at 4°C. Next day the cells were washed with PBS × 3 times and the cells were incubated with secondary antibody (anti-rabbit-Cy3, 1:500, donkey polyclonal, Jackson ImmunoResearch) in PBS at room temperature for 1 h. After the cells were washed with PBS × 3 times, the cell images were captured with EVOS FL (Thermo Fisher Scientific) (Figure 2A).

### Statistical analysis

All the data were analyzed using one-way or two-way analysis of variance (ANOVA) and Student’s *t*-test (2 tailed) for experiments with only two subgroups; a statistical significance was defined as *P* < 0.05. Repeated experiments were analyzed as a randomized complete block design. Means were compared by *post hoc* multiple-comparison test (Tukey’s or Sidak’s), and all values are presented as mean ± SEM and as individual values in dot plots. All the analyses were performed with GraphPad Prism version 8 (GraphPad Software).

### Study approval

All animals were handled and procedures were performed in adherence to the National Institutes of Health *Guide for the Care and Use of Laboratory Animals.* All protocols were approved by the University of Virginia, the University of Tokyo or Nagasaki University Institutional Animal Care and Use Committee.

## Data availability statement

The datasets presented in this study can be found in online repositories. The names of the repository/repositories and accession number(s) can be found below: https://www.ncbi.nlm.nih.gov/, GSE103062 and GSE103058.

## Ethics statement

The animal study was reviewed and approved by University of Virginia, the University of Tokyo or Nagasaki University.

## Author contributions

TI, YN, ST, AB, DR, KI, MN, YW, and MDO designed the research studies. TI, YN, ST, TK, LL, LH, JY, SK, RI (9th author), HN, DF, RiU, SH, RyU, C-HW, and HY conducted the experiments and acquired and analyzed the data. TI, DR, RI (14th author), MN, and MDO wrote the manuscript. All authors contributed to the article and approved the submitted version.

## Footnotes

1
http://humphreyslab.com/SingleCell/

2
http://imagej.nih.gov/ij/

3
http://compbio.mit.edu/cummeRbund/

4
https://software.broadinstitute.org/software/igv/
