# Supplementary material for: Bone marrow stromal cell antigen-1 (CD157) regulated by sphingosine kinase 2 mediates kidney fibrosis
Source: Front Med (Lausanne). 2022 Oct 4;9:993698. doi: 10.3389/fmed.2022.993698 (PMC9576863; doi:10.3389/fmed.2022.993698)

Supplementary Material

**Supplementary Table1. The number of histone acetylation sites**

| H3K9ac | *WT* | *Sphk1^-/-^* | *Sphk2^-/-^* |
| --- | --- | --- | --- |
| Number of acetylation sites | 21430 | 32551 | 34089 |
| Number of genes | 9926 | 10947 | 11091 |

| H3K27ac | *WT* | *Sphk1^-/-^* | *Sphk2^-/-^* |
| --- | --- | --- | --- |
| Number of acetylation sites | 19542 | 14547 | 22465 |
| Number of genes | 5530 | 5019 | 7532 |

**Supplementary Table 2. The list of selected 30 candidate genes related with renal fibrosis**

| Gene name | *WT* | *SphK1^-/-^* | *SphK2^-/-^* |
| --- | --- | --- | --- |
| *Havcr1* | 503.881 | 633.738 | 118.029 |
| *Pdzk1ip1* | 193.743 | 167.248 | 20.3958 |
| *Bst1* | 69.2513 | 84.0208 | 8.43151 |
| *Cdh13* | 57.3541 | 155.29 | 10.0427 |
| *L1cam* | 54.4459 | 45.3611 | 6.95248 |
| *Dcdc2a* | 50.487 | 37.5665 | 6.05082 |
| *Igsf5* | 40.0852 | 38.5272 | 8.88927 |
| *Olfm1* | 37.1044 | 35.5064 | 6.21835 |
| *Agt* | 33.5099 | 46.3409 | 8.27908 |
| *Ntn1* | 33.3354 | 30.5878 | 7.01548 |
| *Pou3f3* | 28.8946 | 26.0993 | 5.86353 |
| *Tshz2* | 23.6499 | 24.0698 | 3.83438 |
| *Limch1* | 15.6841 | 16.0528 | 3.42985 |
| *Slc35f3* | 12.8202 | 12.8387 | 1.58298 |
| *Arhgef16* | 9.8779 | 8.31362 | 1.71332 |
| *Galnt14* | 9.76278 | 11.3559 | 2.00179 |
| *Pitpnm3* | 8.48061 | 11.4621 | 1.47963 |
| *Zic2* | 7.44137 | 9.42822 | 1.39665 |
| *Tmem254b* | 7.0223 | 7.32578 | 0.298666 |
| *Slc35f1* | 5.48198 | 6.70505 | 0.854698 |
| *Ccnjl* | 5.28739 | 5.93895 | 0.948309 |
| *Zic5* | 3.63599 | 4.5168 | 0.613555 |
| *Csmd1* | 2.69362 | 5.36929 | 0.299779 |
| *Rbm47* | 2.4977 | 2.21298 | 0.363405 |
| *Marveld3* | 2.35241 | 2.54933 | 0.330605 |
| *Sigirr* | 2.29259 | 5.37522 | 0.457867 |
| *Timd2* | 1.65154 | 1.85248 | 0.245374 |
| *Erbb4* | 1.61889 | 1.719 | 0.128039 |
| *Gm5833* | 1.59827 | 3.30831 | 0.258472 |
| *Gm853* | 1.58264 | 3.03935 | 0.333686 |

Fragments per kilobase of transcript per million mapped reads (Fpkm)

**Supplementary Table 3. Primer sequences for real-time quantitative PCR**

| *Gapdh*_fwd | ACGGCAAATTCAACGGCACAGTCA |
| --- | --- |
| *Gapdh*_rev | TGGGGGCATCGGCAGAAGG |
| *Acta2 (αSMA)*_fwd | ATTGTGCTGGACTCTGGAGATGGT |
| *Acta2* *(αSMA)_*rev | TGATGTCACGGACAATCTCACGCT |
| *Col3a1*_fwd | TCCTAACCAAGGCTGCAAGATGGA |
| *Col3a1*_rev | ACCAGAATCTGTCCACCAGTGCTT |
| *Vim*_fwd | AGATGGCTCGTCACCTTCGTGAAT |
| *Vim*_rev | TTGAGTGGGTGTCAACCAGAGGAA |
| *Col1a1*_fwd | GAGCGGAGAGTACTGGATCG |
| *Col1a1*_rev | TACTCGAACGGGAATCCATC |
| *Fn1*_fwd | ACCAACCTTAATCCGGGCAC |
| *Fn1*_rev | TCAGAAACTGTGGCTTGCTGG |
| *Cdh13*_fwd | CGCTTCTTCTAGTCGGGCAA |
| *Cdh13*_rev | GCAGGCTGGTGGATGTGTAA |
| *Dcdc2a*_fwd | GCAATGGGGACCCCTTTTTC |
| *Dcdc2a*_rev | GGTGTAGATGTTCCTAACCGC |
| *Tshz2*_fwd | CAGTGGCTCCGTTGCTCAA |
| *Tshz2*_rev | GGAGAGACTCGTTTTCTGCATC |
| *Bst1*_fwd | AGGGACAAGTCACTGTTCTGG |
| *Bst1*_rev | AACTTTGCCATACAGCACGTC |
| *Kim-1* (*Havcr1*)_fwd | ACATATCGTGGAATCACAACGAC |
| *Kim-1* (*Havcr1*)_rev | ACTGCTCTTCTGATAGGTGACA |
| *Ntn1*_fwd | GTGGAGGAACCGGAAGACTG |
| *Ntn1*_rev | TTGTCGGCCTTCAGGATGTG |
| *Olfm1*_fwd | GGTGGAGGAGAGCCATAAGC |
| *Olfm1*_rev | TCTTCAGTCACTGGCGCATT |
| *L1cam*_fwd | AAAGGTGCAAGGGTGACATTC |
| *L1cam*_rev | TCCCCACGTTCCTGTAGGT |
| *Igsf5*_fwd | CTCAGTGCAAGTCATGGGGAC |
| *Igsf5*_rev | CCAGGAAATATCCGGGAGTGA |
| *Agt*_fwd | TCTCCTTTACCACAACAAGAGCA |
| *Agt*_rev | CTTCTCATTCACAGGGGAGGT |
| *Slc35f3*_fwd | CCCATTGTACTACGCAGGACA |
| *Slc35f3*_rev | AACCGACAGCATTCCCTGTAT |
| *Limch1*_fwd | ATGTTTGACATGCGGTGTGAG |
| *Limch1*_rev | ATCTTGCCATTTGTCGTCCTC |
| *Arhgef16*_fwd | GCCATCTTCGAGATCCTCACT |
| *Arhgef16*_rev | GGTGGTGGTGTTCCGTCTG |
| *Pdzk1ip1*_fwd | TTGGCCTTCAGTCTGCTCG |
| *Pdzk1ip1*_rev | CAGAAGTGGTTGACGGCGAA |
| *Pou3f3*_fwd | AGCTGGAAAAGGAGGTCGTG |
| *Pou3f3*_rev | CCTGCGAGTAGACATCGTCC |
| *Sphk2*_fwd | GCACGGCGAGTTTGGTTC |
| *Sphk2*_rev | GAGACCTCATCCAGAGAGACTAG |

## *Acta2 (αSMA)*, actin, alpha 2, smooth muscle, aorta; *Agt*, angiotensinogen; *Arhgef16*, Rho guanine nucleotide exchange factor (GEF) 16; *Bst1*, bone marrow stromal cell antigen 1; *Cdh13*, cadherin 13; *Col1a1*, collagen, type I, alpha 1; *Col3a1*, collagen, type III, alpha 1; *Dcdc2a*, doublecortin domain containing 2a; *Fn1*, fibronectin 1; *Gapdh*, glyceraldehyde 3-phosphate dehydrogenase; *Havcr1 (Kim1)*, hepatitis A virus cellular receptor 1; *Igsf5*, immunoglobulin superfamily, member 5; *L1cam*, L1 cell adhesion molecule; *Limch1*, LIM and calponin homology domains 1; *Ntn1*, netrin 1; *Olfm1*, olfactomedin 1; *Pdzk1ip1*, PDZK1 interacting protein 1; *Pou3f3*, POU domain, class 3, transcription factor 3; *Slc35f3*, solute carrier family 35, member F3; *Tshz2*, teashirt zinc finger family member 2; *Vim*, vimentin.

**Supplementary Table 4. Primer sequences for ChIP-qPCR**

| Negative control at *Bst1*_fwd | AGCCCACGTTCTGAGACAAG |
| --- | --- |
| Negative control at *Bst1*_rev | GCAGTCTACGAACGGAGAGG |
| Promoter at *Bst1*_fwd | TGTGGCTATGGCTGTCCTTG |
| Promoter at *Bst1*_rev | ATGCTCTGGAGGTGAGGAGT |

**Supplementary Table 5. Primer sequences for tail genotyping**

| Sphk1-1 | TGTCACCCACGAACCTGCTGTCCCTGCACA |
| --- | --- |
| Sphk1-2 | AGAAGGCACTGGCTCCTCCAGAGGAACAAG |
| Sphk1-3 | TCGTGCTTTACGGTATCGCCGCTCCCGATT |
| Sphk2-1 | GCACCCAGTGTGAATCGAGC |
| Sphk2-2 | TCTGGAGACGGGCTGCTTTA |
| Sphk2-3 | CGCTATCAGGACATAGCGTT |
| Bst1-1 | ACAAAGGAAGAAAAGGAAAGACAAAACAGG |
| Bst1-2 | AGGAAGATGCTCTGGAGGTGAGGAGTGGTA |
| Bst1-3 | CTGAAGAGCTTGGCGGCGAATGGGCTGACC |

##

**Supplementary Figure 1.** **The kidney of *Sphk1^-/-^* mice was injured after unilateral IRI.**

Homozygous *Sphk1^-/-^* mice were crossed with C57BL/6 WT mice to generate littermate *Sphk1^+/+^* WT controls and *Sphk1^+/-^* heterozygotes. Nephrectomy (right) was performed 13 days after renal unilateral (left) ischemia-reperfusion injury (UniIRI; 26 minutes ischemia). One day later mice were euthanized (Euth) and function of the remaining kidney was evaluated by measuring plasma creatinine. *Sphk1^-/-^* mice had the same degree of injury as WT (*Sphk1^+/+^*mice) and heterozygous (*Sphk1^+/-^* mice) littermates. n=7-9. Data were analyzed using one-way ANOVA. Means were compared by post hoc multiple-comparison test (Tukey’s). This figure and Supplemental Figure S2 demonstrate the effect of *Sphk1* and *Sphk2* deletion on renal fibrosis after uniIRI relative to the appropriate WT littermate *Sphk1^+/+^* and *Sphk2^+/+^* control mice. Data from these WT littermate *Sphk1^+/+^* and *Sphk2^+/+^* control mice were then combined and used as “WT” controls for the experiments in Figure 1c-e and Figure 2c, which compares WT, *Sphk1^-/-^* and *Sphk2^-/-^* mice.

**Supplementary Figure 2.** **Kidney function of *SphK2^-/-^* mice was preserved after unilateral IRI.**

Homozygous *Sphk2^-/-^* mice were crossed with C57BL/6 WT mice to generate littermate *Sphk2^+/+^* WT controls and *Sphk2^+/-^* heterozygotes. Nephrectomy (right) was performed 13 days after renal unilateral (left) ischemia-reperfusion injury (IRI; 26 minutes ischemia). One day later function of the remaining kidney was evaluated by measuring plasma creatinine. n=5-7. Plasma creatinine was significantly lower (indicating preserved kidney function) in *Sphk2^-/-^* mice compared to WT (*SphK2^+/+^* mice) and heterozygous (*SphK2^+/-^* mice) littermates. Data were analyzed using one-way ANOVA. Means were compared by post hoc multiple-comparison test (Tukey’s). Supplemental Figure S1 and S2 demonstrate the effect of *Sphk1* and *Sphk2* deletion on renal fibrosis after uniIRI relative to the appropriate WT littermate *Sphk1^+/+^* and *Sphk2^+/+^* control mice. Data from these WT littermate *Sphk1^+/+^* and *Sphk2^+/+^* control mice were then combined and used as “WT” controls for the experiments in Figure 1c-e and Figure 2c, which compares WT, *Sphk1^-/-^* and *Sphk2^-/-^* mice.

**
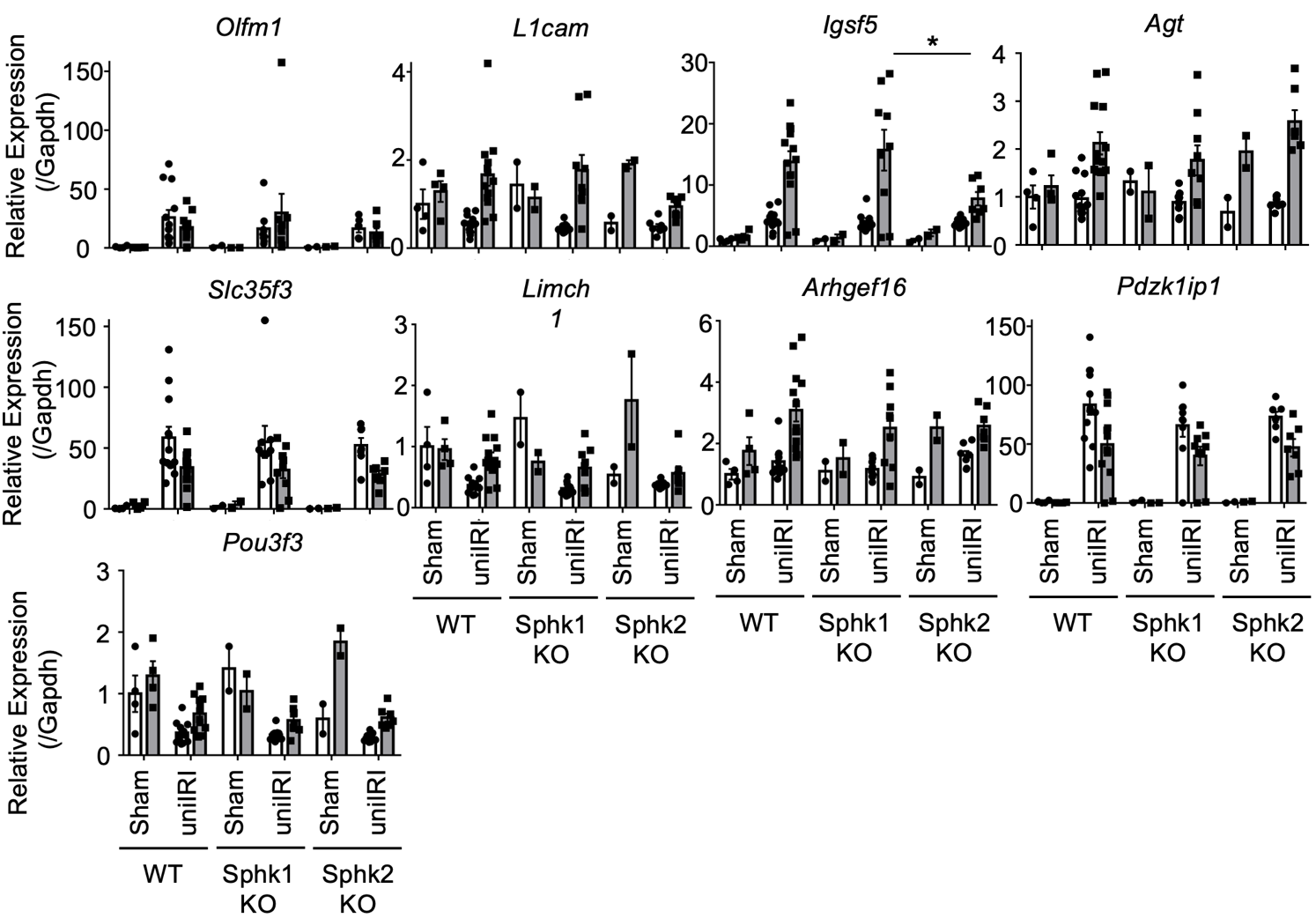
**
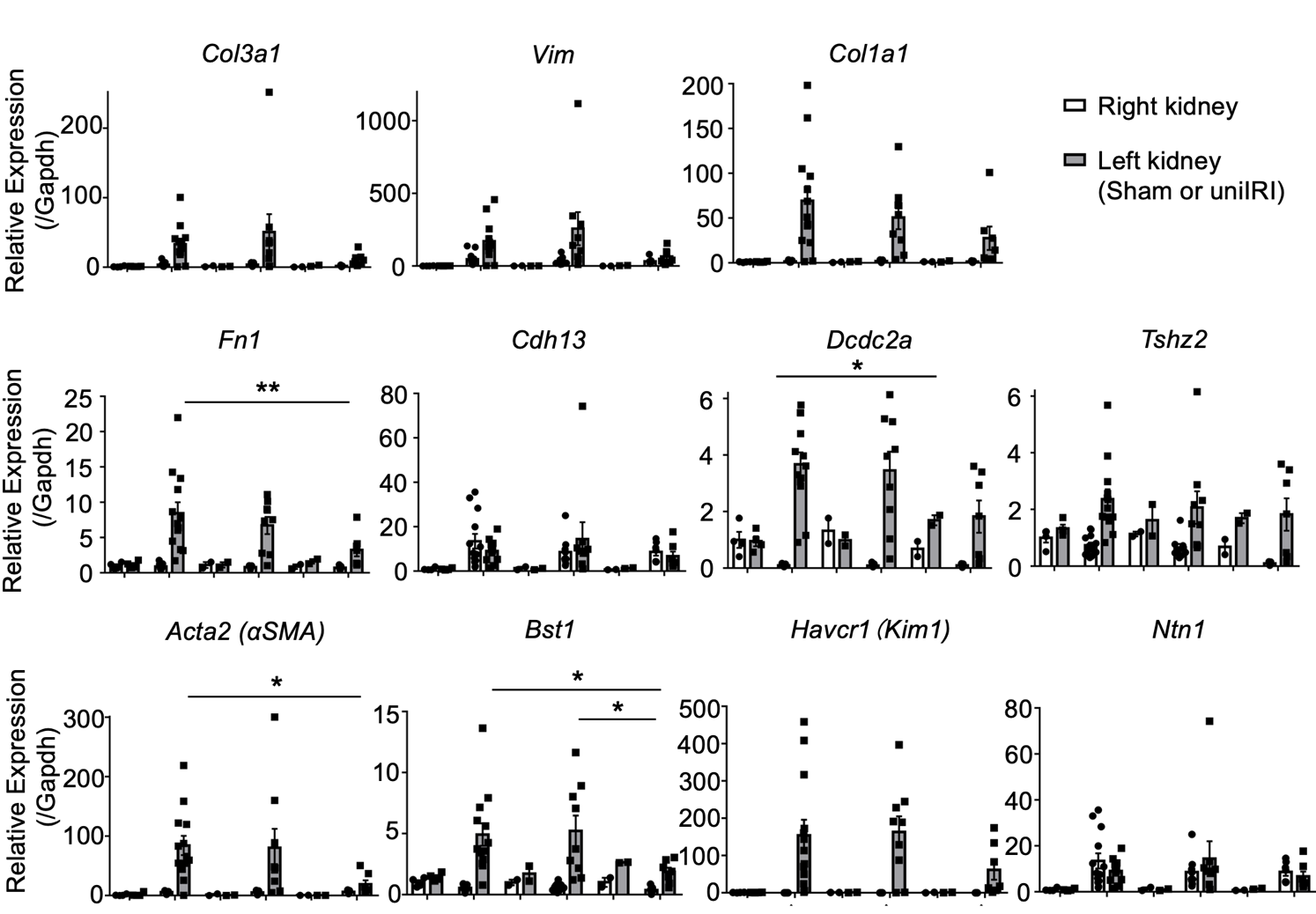


**Supplementary Figure 3.** **The detailed data of *in vivo* screening.**

The detailed data for Figure 2c. Nephrectomy (right) was performed 13 days after renal unilateral (left) ischemia-reperfusion injury (sham or uniIRI; 26 minutes ischemia). One day later the remaining (left, injured) kidney was obtained after the mice were euthanized. RNA was isolated from the kidneys, then real time PCR was performed using the primers (Supplemental Table S3) for fibrosis-related genes and the top half of genes selected by the combination of RNA-seq and ChIP-seq shown in Figure 2b and Table S2. n=2-4 for sham and n=13 for *WT*, n=9 for *Sphk1^-/-^* (Sphk1 KO) and n=7 for *Sphk2^-/-^* (Sphk2 KO). Data were analyzed using two-way ANOVA. Means were compared by *post hoc* multiple comparison test (Sidak's). * *P*< 0.05, ** *P*<0.01 and *** *P*<0.001. *Acta2 (αSMA)*, actin, alpha 2, smooth muscle, aorta; *Agt*, angiotensinogen; *Arhgef16*, Rho guanine nucleotide exchange factor (GEF) 16; *Bst1*, bone marrow stromal cell antigen 1; *Cdh13*, cadherin 13; *Col1a1*, collagen, type I, alpha 1; *Col3a1*, collagen, type III, alpha 1; *Dcdc2a*, doublecortin domain containing 2a; *Fn1*, fibronectin 1; *Havcr1 (Kim1)*, hepatitis A virus cellular receptor 1; *Igsf5*, immunoglobulin superfamily, member 5; *L1cam*, L1 cell adhesion molecule; *Limch1*, LIM and calponin homology domains 1; *Ntn1*, netrin 1; *Olfm1*, olfactomedin 1; *Pdzk1ip1*, PDZK1 interacting protein 1; *Pou3f3*, POU domain, class 3, transcription factor 3; *Slc35f3*, solute carrier family 35, member F3; *Tshz2*, teashirt zinc finger family member 2; *Vim*, vimentin.

**Supplementary Figure 4.** **Images of picrosirius red-stained kidney sections from *Bst1^+/+^* and *Bst1^-/-^* mice.**

Representative photographs of picrosirius red staining of kidney sections. Nephrectomy (right) was performed 13 days after renal unilateral (left) ischemia-reperfusion injury (UniIRI; 26 minutes ischemia) and kidney injury was evaluated one day later (same mice as in Figure 5). *Bst1^+/+^* and *Bst1^-/-^* mice were used. Quantitative analysis is shown in Supplemental Figure S5. Scale bar = 1 mm in whole kidney, 200 μm in polarized view.

**Supplementary Figure 5.** ***Bst1^-/-^* mice develop less fibrosis after unilateral IRI.**

Nephrectomy (right) was performed 13 days after renal unilateral (left) ischemia-reperfusion injury (UniIRI; 26 minutes ischemia) and kidney injury was evaluated one day later. *Bst1^+/+^* and *Bst1^-/-^* mice were used. *Bst1*-deficient mice developed less renal fibrosis as shown by scored data (collagen deposition as percent of total kidney surface area) from polarized picrosirius red photographs (Supplemental Figure S4). n=7-10. Data were analyzed using two-way ANOVA, followed by *post hoc* multiple-comparison test (Tukey’s). ** *P*<0.01.

**
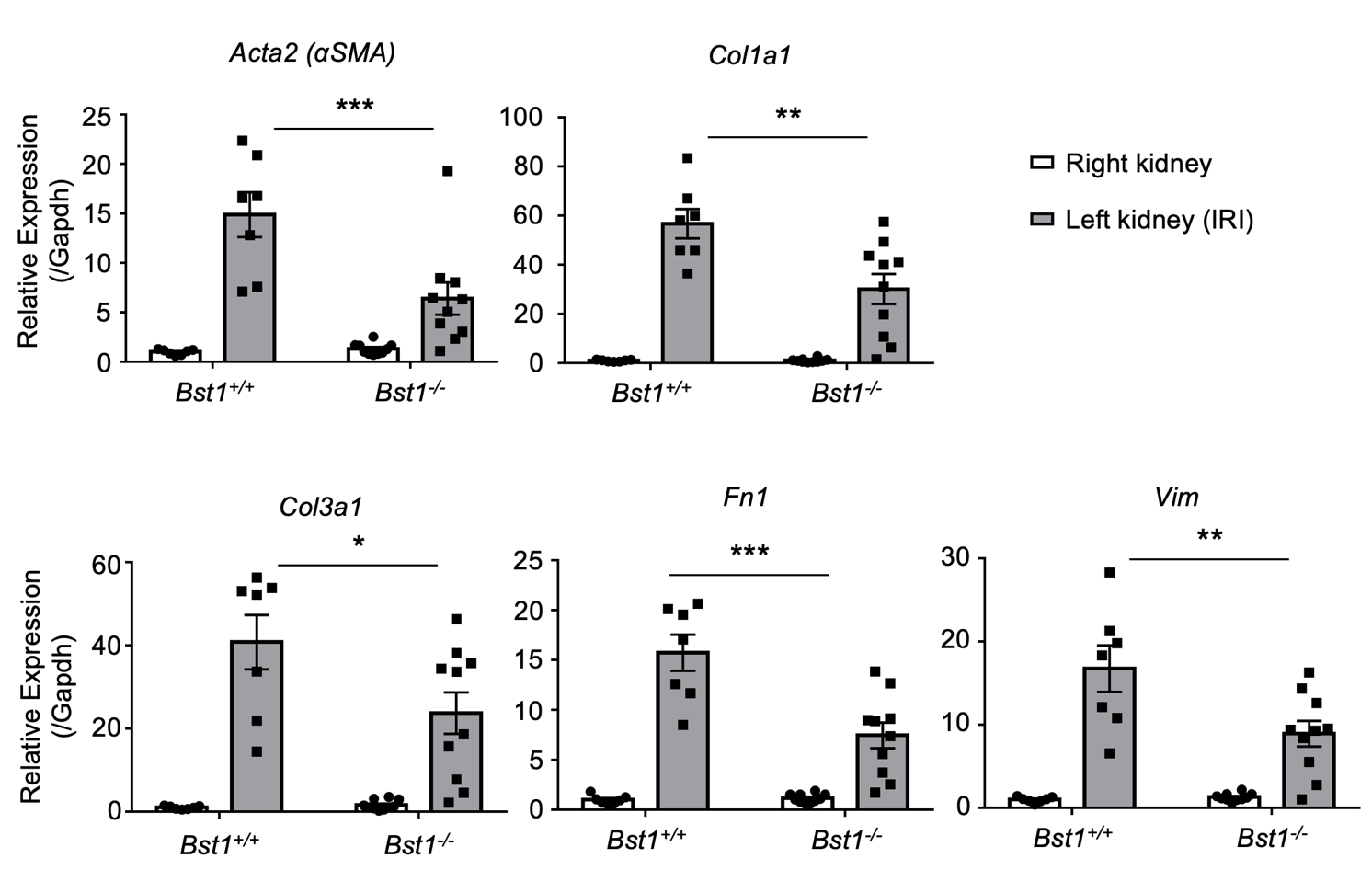
Supplementary Figure 6.** **Expression of fibrosis markers in the kidney after unilateral IRI were suppressed in *Bst1^-/-^* mice.**

The detailed data for Figure 5c. Nephrectomy (right) was performed 13 days after renal unilateral (left) ischemia-reperfusion injury (IRI; 26 minutes ischemia). One day later the remaining (left) kidney was obtained after the mice were euthanized. RNA was isolated from the kidneys, then real time PCR was performed. Data were analyzed with Student’s t-test (2 tailed). * *P*< 0.05, ** *P*<0.01 and *** *P*<0.001. n = 7-10.

**Supplementary Figure 7.** **Kidney function of *Bst1^-/-^* mice was preserved after unilateral IRI.**

Homozygous *Bst1^-/-^* mice were crossed with C57BL/6 WT mice to generate littermate *Bst1^+/+^* WT controls and *Bst1^+/-^* heterozygotes. Nephrectomy (right) was performed 13 days after renal unilateral (left) ischemia-reperfusion injury (IRI; 26 minutes ischemia). One day later the remaining kidney function was evaluated by plasma creatinine. n=7-10. Data were analyzed using one-way ANOVA. Means were compared by post hoc multiple-comparison test (Tukey’s). *** *P*<0.001.

**Supplementary Figure 8. Genotyping of Sphk1^-/-^, Sphk2^-/-^, Bst1^-/-^ mice.**


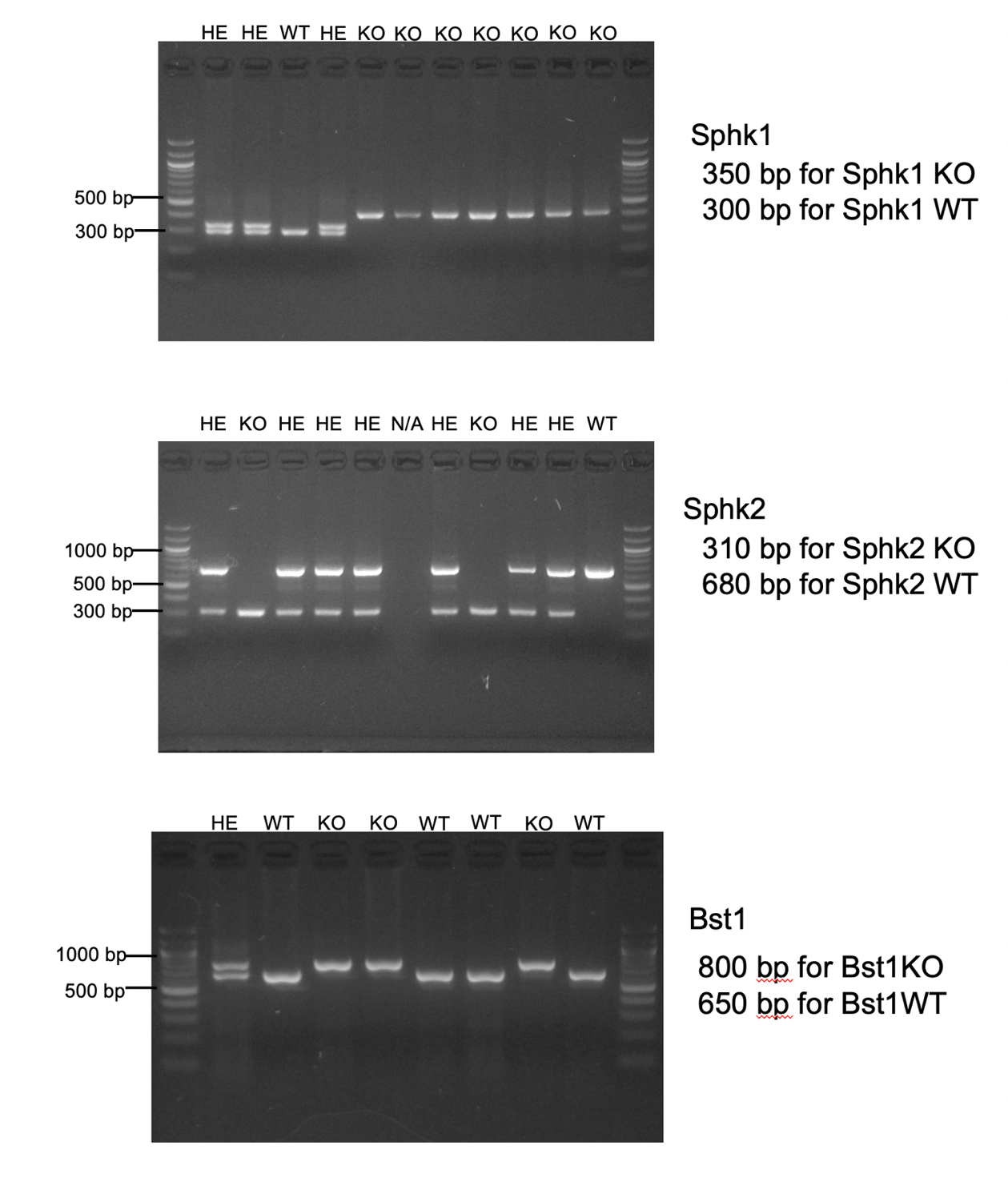

Supplement: Supplementary file 1 [file Data_Sheet_1.docx]
